# Step-by-Step Cadaver Dissection and Surgical Technique for Compartmental Tongue and Floor of Mouth Resection

**DOI:** 10.3389/fonc.2021.613945

**Published:** 2021-04-23

**Authors:** Alberto Grammatica, Cesare Piazza, Marco Ferrari, Vincenzo Verzeletti, Alberto Paderno, Davide Mattavelli, Alberto Schreiber, Davide Lombardi, Enrico Fazio, Luca Gazzini, Giovanni Giorgetti, Barbara Buffoli, Luigi Fabrizio Rodella, Piero Nicolai, Luca Calabrese

**Affiliations:** ^1^ Department of Otorhinolaryngology – Head and Neck Surgery, University of Brescia, Brescia, Italy; ^2^ Department of Otorhinolaryngology – Head and Neck Surgery, University of Padua, Padua, Italy; ^3^ Department of Otorhinolaryngology – Head and Neck Surgery, “San Maurizio” Hospital, Bolzano, Italy; ^4^ Department of Clinical and Experimental Sciences, Section of Anatomy and Physiopathology, University of Brescia, Brescia, Italy

**Keywords:** tongue cancer, oral cavity, compartmental tongue surgery, cadaver dissection, surgical technique

## Abstract

**Background:**

The aim of oral cancer surgery is tumor removal within clear margins of healthy tissue: the latter definition in the literature, however, may vary between 1 and 2 cm, and should be intended in the three dimensions, which further complicates its precise measurement. Moreover, the biological behavior of tongue and floor of mouth cancer can be unpredictable and often eludes the previously mentioned safe surgical margins concept due to the complexity of tongue anatomy, the intricated arrangements of its intrinsic and extrinsic muscle fibers, and the presence of rich neurovascular and lymphatic networks within it. These structures may act as specific pathways of loco-regional tumor spread, allowing the neoplasm to escape beyond its visible macroscopic boundaries. Based on this concept, in the past two decades, compartmental surgery (CS) for treatment of oral tongue and floor of mouth cancer was proposed as an alternative to more traditional transoral resections.

**Methods:**

The authors performed three anatomical dissections on fresh-frozen cadaver heads that were injected with red and blue-stained silicon. All procedures were documented by photographs taken with a professional reflex digital camera.

**Results:**

One of these step-by-step cadaver dissections is herein reported, detailing the pivotal points of CS with the aim to share this procedure at benefit of the youngest surgeons.

**Conclusions:**

We herein present the CS step-by-step technique to highlight its potential in improving loco-regional control by checking all possible routes of tumor spread. Correct identification of the anatomical space between tumor and nodes (T-N tract), spatial relationships of extrinsic tongue muscles, as well as neurovascular bundles of the floor of mouth, are depicted to improve knowledge of this complex anatomical area.

## Introduction

Compartmental surgery (CS) has emerged in the last decade as a promising approach for treatment of locally advanced cancers of the tongue and floor of mouth. The term “compartmental” has been borrowed from the field of sarcoma surgery which typically aims to remove an entire anatomical compartment defined as an entire group of muscle fibers, vascular, lymphatic and neural bundles along with the overlaying fascial system that usually drive tumor growth and direction, following the spatial orientation of these structures (1, 2). First conceived and proposed by Calabrese and coworkers in 2009, CS allows better oncological outcomes compared to traditional wide-margin (1-2 cm) resections, in terms of both local and loco-regional control (3, 4). This standardized technique involves the en-bloc resection of one hemitongue and related floor of mouth *via* pull-through or transmandibular approaches, clearing the neck lymph nodes in continuity with the “tumor-nodes” (T-N) tract. This has been demonstrated to achieve optimal loco-regional control, while not substantially impacting functional outcomes and residual quality of life (5–7).

The ideal indications for CS are represented by tongue and/or floor of mouth cancers with a clinical/radiological depth of infiltration (DOI) ≥10 mm (as ascertained by preoperative MR or CT scans with contrast administration). The importance of a DOI threshold ≥10 mm for tongue cancer has been demonstrated to be crucial in various clinical and anatomical studies, clearly showing that the boundary between the vast majority of intrinsic and extrinsic muscle fibers in such a complex anatomy mainly occurs at this depth (8–11). However, such a parameter cannot be considered an absolute cut-off since the thickness of the intrinsic tongue musculature can vary between different patients, and there is still a relatively large confidence interval in DOI quantification by imaging. The abrupt change of the 3D-spatial arrangement between extrinsic and intrinsic muscular fibers at this level is, however, fundamental for the philosophy at the basis of CS, since it is conceivable that the tumor from this location would start spreading following directions of the connective framework, tending to reach the bone insertions of extrinsic tongue muscles (i.e. mandible, hyoid, and styloid process). Moreover, vessels, lymphatics, and nerves travelling within the paramedian and lateral tongue septa are easily involved when the tumor reaches a DOI of 10 mm in the tongue and floor of the mouth, and play a paramount role in clinical and pathological tumor behavior (12).

We herein present the CS technique in a step-by-step fashion, based on a fresh cadaver dissection focusing on the most valuable anatomical details, in order to make it possible for the reader to reproduce the procedure in an oncologically safe way in routine clinical practice.

## Materials, Equipment and Methods

Three fresh-frozen cadaver heads (Medcure^®^, Portland, Oregon, USA) were dissected in the Laboratory of Anatomy at the University of Brescia, Italy. The arterial and venous systems had been previously injected with red and blue-stained silicon, respectively. A complete set of head and neck surgical instrumentation was available for the dissection. All the anatomical procedures were recorded by a VITOM 3D (Karl Storz^®^, Tuttlingen, Germany) or a 4K endoscope (Olympus^®^, Tokyo, Japan) for academic purposes. The photographs of all anatomical dissections for each step were taken using a reflex digital camera with a 12.3 megapixel resolution (Nikon D300, Nikon, Japan) coupled with a 60 mm F/2 Macro 1:1 fixed focal length lens, mounted on a tripod.

## Results

The best surgical specimen both for quality of tissues and preservation of anatomical details was selected and used for presentation of this step-by-step anatomical/surgical dissection.

### Step 1 - Surgical Field Preparation and Level IB Anatomy

**Figure 1:**
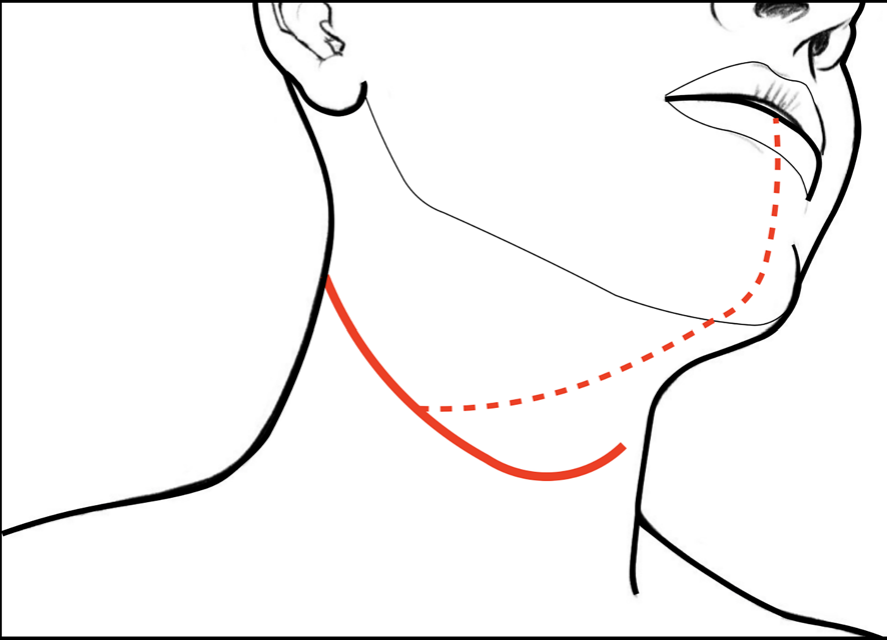
Skin incisions for CS of the tongue and floor of mouth: the continuous line represents that usually followed in case of pull-through approach and unilateral neck dissection (while tracheostomy is performed *via* a separated caudal stab wound); dotted line indicates the incision for the transmandibular approach.

Skin incision is performed following a horizontal crease, usually extending it from the mastoid tip to the thyroid notch or thyro-hyoid membrane, crossing the midline in order to allow appropriate bilateral clearance of submental lymph nodes (level IA) (**Figure 1**, continuous line). If mandibulotomy is needed to reach tumors with massive posterior tongue involvement or associated trismus, skin incision can be accordingly modified by extending it to the mandibular symphysis and lower vermilion (**Figure 1**, dotted line).

A subplatismal cervical flap is raised with exposure of the body of the mandible, paying attention to preserve the mandibularis branch of the VII cranial nerve which runs in a plane deep to the superficial cervical fascia. The procedure usually starts by clearing lymph nodes according to clinical needs (selective neck dissection levels I-III, or levels I-IV, or modified radical or radical neck dissections). As a consequence, the internal jugular vein (IJV) and common carotid artery (CCA) with its bifurcation are fully visible when retracting the sternocleidomastoid muscle (SCM). The external carotid artery (ECA) with its collateral branches is also in full view. Thyroid and occipital arteries (the latter resected) and their anatomical relationships with the XII cranial nerve can be easily appreciated.

**Figure 2:**
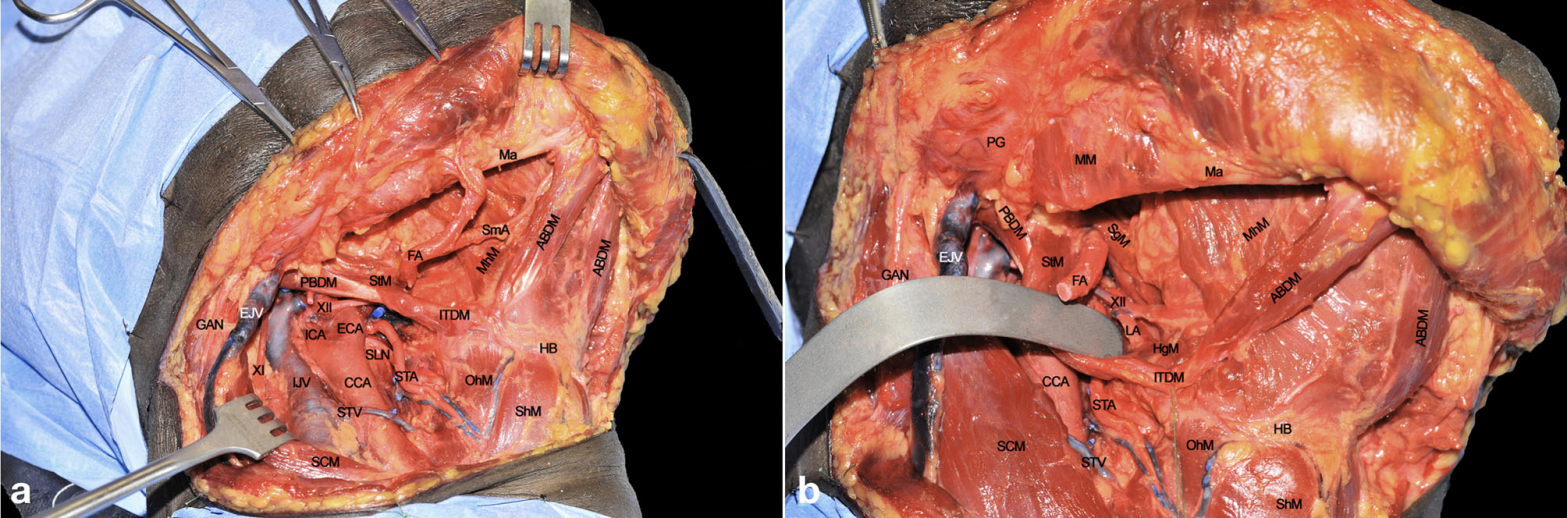
**(A, B)** ABDM, anterior belly of digastric muscle; CCA, common carotid artery; ECA, external carotid artery; EJV, external jugular vein; FA, facial artery; GAN, great auricular nerve; HB, hyoid bone; HgM, hyoglossus muscle; ICA, internal carotid artery; IJV, internal jugular vein; ITDM, intermediate tendon of digastric muscle; LA, lingual artery; Ma, mandible; MhM, mylohyoid muscle; OhM, omohyoid muscle; PBDM, posterior belly of digastric muscle; PG, parotid gland; SCM, sternocleidomastoid muscle; SgM, styloglossus muscle; ShM, sternohyoid muscle; SLN, superior laryngeal nerve; SmA, submental artery; STA, superior thyroid artery; StM, stylohyoid muscle; STV, superior thyroid vein; XI, spinal accessory nerve; XII, hypoglossal nerve.

The digastric muscle with its anterior (ABDM) and posterior bellies (PBDM) is skeletonized to correctly delimitate the inferior borders of the submandibular space. The submandibular gland and adjacent fat tissue containing lymph nodes of level IB is then dissected from the surrounding tissues and detached from the mylohyoid muscle (MhM), which represents the deep plane of dissection. Facial artery (FA) and its submental branch (SmA) are found herein and, whenever possible, preserved (**Figure 2A**).

*Surgical tips and tricks*

- Skin incision in males should be placed caudally to the margin of the beard to make shaving easier in the postoperative period. Usually, putting it in a skin crease minimizes the ensuing aesthetic impact. Great care should be applied to maintain surgical field of the neck separate from the tracheostomy site, thus reducing the risk of contamination and wound infection.
- The facial vessels (artery and vein) are dissected and preserved as long as possible since they represent the first option for subsequent micro-anastomoses during the reconstructive phase.
- The patient is positioned supine with the head extended and turned in order to expose the affected neck side.
- In short necks, it is advisable to put a roll under the patient’s shoulders to further improve neck extension.

### Step 2 - Submandibular Space Dissection and Lingual Artery Exposure

The FA is ligated to clearly expose the submandibular space. The MhM is completely skeletonized, identifying its bony attachment to the mandible and hyoid bone. The intermediate tendon of the digastric muscle (ITDM) is pulled down along with the stylohyoid muscle (StM) in order to highlight the lingual artery (LA) and the XII cranial nerve. This space, also known as Pirogov’s triangle, is bounded by the ITDM, posterior margin of the MhM, and XII cranial nerve cranially. The LA is clearly visible close to its branching point from the ECA while, after 1-2 cm, it lays deep to the hyoglossus muscle (HgM) (**Figure 2B**).

### Step 3 - Hypoglossal and Lingual Nerve Identification

**Figure 3:**
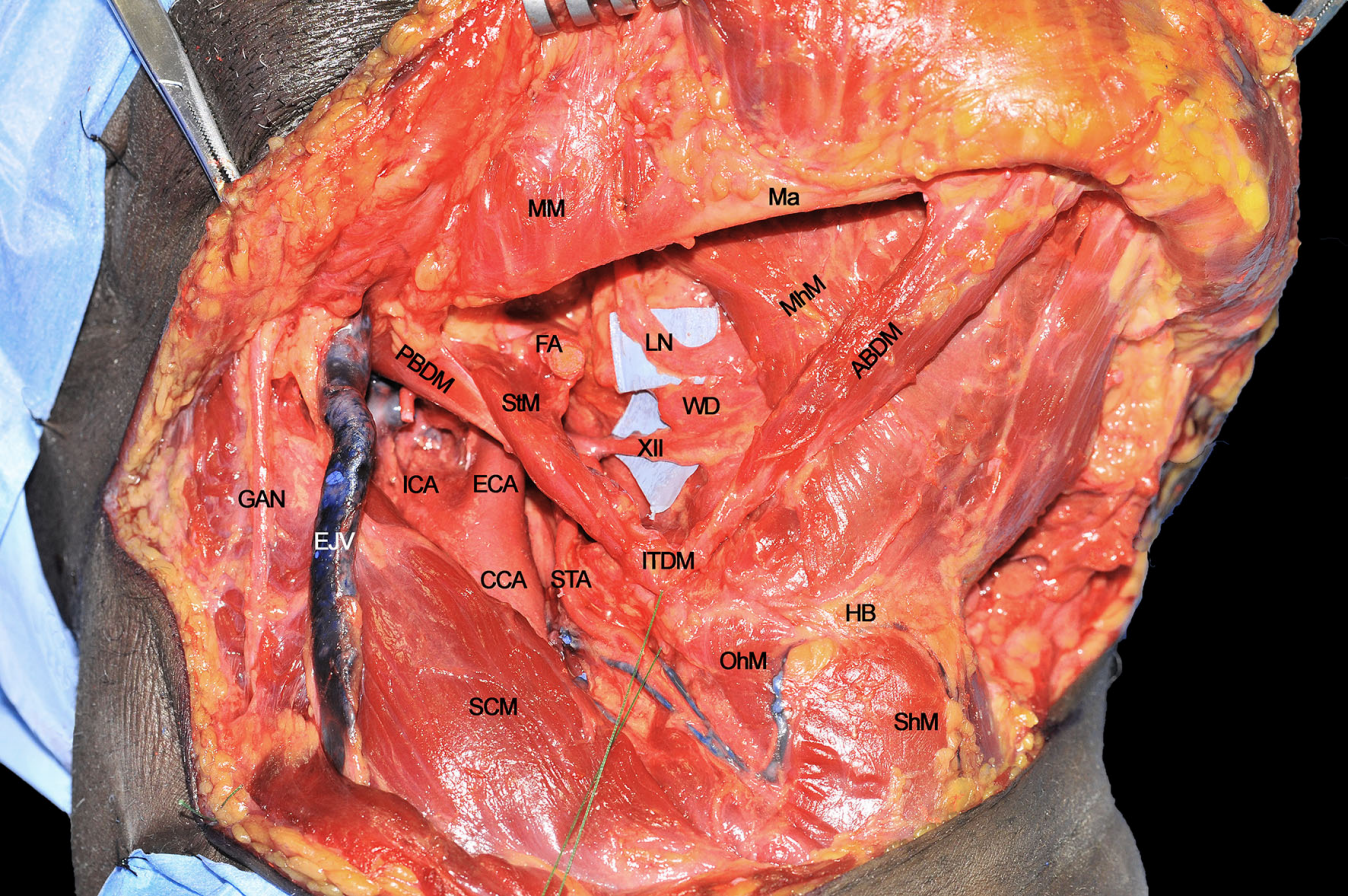
ABDM, anterior belly of digastric muscle; CCA, common carotid artery; ECA, external carotid artery; EJV, external jugular vein; FA, facial artery; GAN, great auricular nerve; HB, hyoid bone; ICA, internal carotid artery; ITDM, intermediate tendon of digastric muscle; LN, lingual nerve; Ma, mandible; MhM, mylohyoid muscle; MM, masseter muscle; OhM, omohyoid muscle; PBDM, posterior belly of digastric muscle; SCM, sternocleidomastoid muscle; ShM, sternohyoid muscle; STA, superior thyroid artery; StM, stylohyoid muscle; WD, Warthon’s duct; XII, hypoglossal nerve.

Once the inferior aspect of the compartment has been delimited, the next step is to identify the two major neural structures potentially acting as routes for tumor spread: the XII cranial nerve and the lingual nerve (LN). The former is identified from posterior to anterior crossing the ECA, running deeply to the PBDM and StM, and superficially to the HgM (hidden by the silicone background) and LA. Two centimeters cranially and anteriorly, running deeply to the MhM, the LN can be found in close relationship with the sublingual gland and floor of mouth mucosa. The XII cranial nerve must be followed and sectioned, preserving, if feasible, the emergence of its descending loop with the cervical plexus to maintain the strap muscles innervation (**Figure 3**). Dissection continues deeply, in a caudo-cranial way by detaching the HgM from its hyoid insertions, thus exposing and ligating the LA in close proximity to the greater cornu of the hyoid bone.

*Surgical tips and tricks*

- Frozen sections should be sent from the cranial stump of the hypoglossal nerve, especially in the presence of advanced cancers massively infiltrating the MhM and HgM.
- Identification of the HgM is made easier by gripping the hyoid bone with Ellis forceps and pulling it laterally.
- The LA must be ligated and frozen sections sent for possible vascular invasion evaluation, especially when floor of the mouth gross involvement is detected. This maneuver will also greatly reduce bleeding during subsequent tongue resection. The LA can be also used as an alternative to the FA as a donor vessel for microvascular anastomoses.
- The anatomical preservation of the descending loop of the XII cranial nerve may improve swallowing in the postoperative period.

### Step 4 - Mylohyoid and Hyoglossus Muscles Detachment

**Figure 4:**
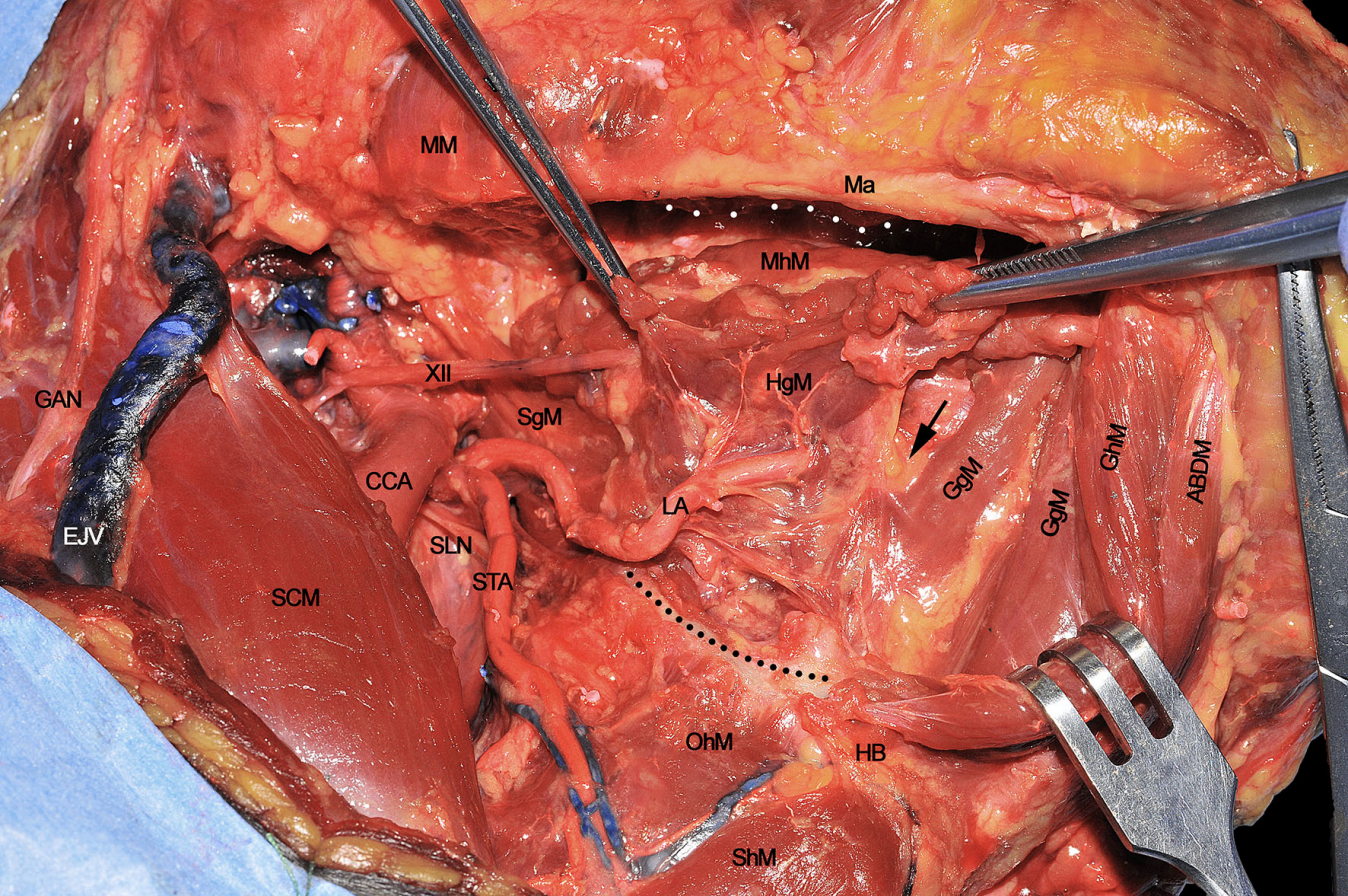
ABDM, anterior belly of digastric muscle; CCA, common carotid artery; EJV, external jugular vein; GAN, great auricular nerve; GgM, genioglossus muscle; GhM, geniohyoid muscle; HB, hyoid bone; HgM, hyoglossus muscle; LA, lingual artery; Ma, mandible; MhM, mylohyoid muscle; MM, masseter muscle; OhM, omohyoid muscle; SCM, sternocleidomastoid muscle; SgM, styloglossus muscle; ShM, sternohyoid muscle; SLN, superior laryngeal nerve; STA, superior thyroid artery; XII, hypoglossal nerve. White dotted line, mylohyoid line of the mandible. Black dotted line, great hyoid cornu. Black arrow, paramedian septum of the tongue.

Once the above mentioned neurovascular structures have been identified, the dissection proceeds delimitating the T-N tract from caudal to cranial following the bony attachments of the different muscular structures. In this specific case, the digastric muscle has been removed to better expose the extrinsic lingual muscular compartment (even though not necessarily done in all patients treated by CS). The LA, clearly visible at the level of its branching from the ECA, is followed through the HgM (grabbed with forceps), and detached from its hyoid insertions (black dotted line). Cranially, the MhM has been detached from the mylohyoid line (white dotted line) on the internal aspect of the mandibular body, while the LN is exposed and sectioned as cranial as possible. Anteriorly, the ABDM is retracted medially to show the fatty median raphe between the left and right genioglossus muscles (GgM). Anteriorly and superficially the geniohyoid muscle (GhM) and deeply both the GgMs are well visible. Lateral to the right GgM the fat-containing paramedian septum is recognizable (black arrow) (**Figure 4**).

*Surgical tips and tricks*

- If not oncologically required, it is not advisable to completely skeletonize the hyoid bone, especially in previously irradiated patients, to reduce the risk of postoperative osteonecrosis.
- Conversely, when detaching the MhM from the inner mandibular surface, it is advisable to use monopolar cautery, taking care to cut the fibers directly over the bone in a subperiosteal plane (a blunt dissector can be also used to assist the surgeon in this crucial maneuver). This is of paramount importance, especially when managing tumors involving the antero-lateral floor of the mouth, with close relationship to the mandible, even without radiological signs of erosion, in order to assess a possible limited cortical bone or periosteal neoplastic invasion.

### Step 5 - Approach to the Midline in the Submental Area

**Figure 5:**
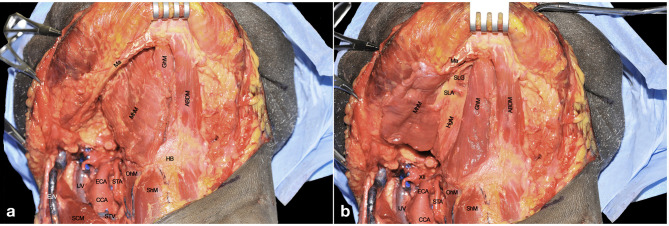
**(A, B)** ABDM, anterior belly of digastric muscle; CCA, common carotid artery; ECA, external carotid artery; EJV, external jugular vein; GhM, geniohyoid muscle; HB, hyoid bone; HgM, hyoglossus muscle; IJV, internal jugular vein; Ma, mandible; MhM, mylohyoid muscle; OhM, omohyoid muscle; SCM, sternocleidomastoid muscle; ShM, sternohyoid muscle; SLA, sublingual artery; SlG, sublingual gland; STA, superior thyroid artery; STV, superior thyroid vein; XII, hypoglossal nerve.

Once the lateral compartment has been prepared, the head is turned to a neutral position while extended in order to fully expose the region from the hyoid bone up to the mandible. The midline is found by sectioning the MhM in between the two ABDMs. The section line (**Figure 5A**) should be traced from the body of the hyoid bone to the genial tubercles of the mandible. In the present cadaver dissection, the right digastric muscle has been removed to better expose the underlying anatomy, while it is easily recognizable on the left side. Particular care should be given to the MhM dissection in order to properly find the midline between the GhMs, just beneath its deeper surface. This step is crucial when treating tumors massively involving the anterior floor of mouth, to exclude possible neoplastic spread through the midline into the opposite compartment. Moreover, adequate intraoperative assessment of the MhM deeper portion is mandatory, due to the possible presence of in-transit nodal metastases and/or tumor satellitosis at this level.

Once the dissection of the MhM has been completed along the midline, this can be retracted laterally to fully expose the underlying GhM. This leads to optimal visualization of the paramedian lingual septum, which contains lymphatic vessels and nodes, neural bundles of the LN, the sublingual gland (SlG), and branches of the sublingual artery (SLA) (**Figure 5B**). This space is of special relevance when performing CS, since it represents one of the most important routes for tumor spread.

**Figure 6:**
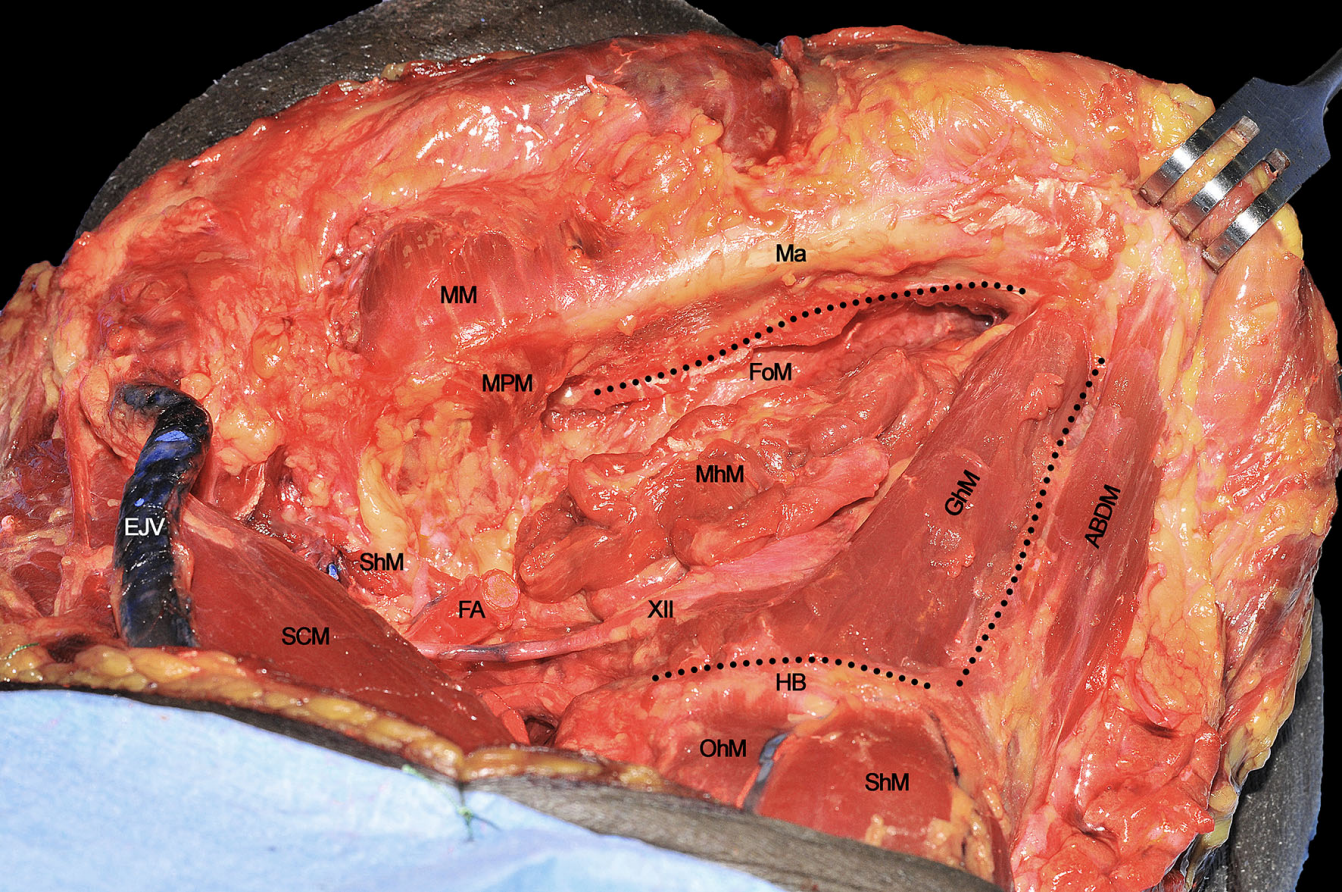
ABDM, anterior belly of digastric muscle; EJV, external jugular vein; FA, facial artery; FoM, floor of mouth; GhM, geniohyoid muscle; HB, hyoid bone; Ma, mandible; MhM, mylohyoid muscle; MM, masseter muscle; MPM, medial pterygoid muscle; OhM, omohyoid muscle; SCM, sternocleidomastoid muscle; ShM, sternohyoid muscle; StM, stylohyoid muscle; XII, hypoglossal nerve. Black dotted line, boundaries of the anatomical area containing the T-N tract.

The anatomical area in which the T-N tract can be located is defined by the following boundaries: the inferior aspect of the floor of mouth (FoM) mucosa cranially, the GhM anteriorly, and the HB caudally (black dotted line) (**Figure 6**).

*Surgical tips and tricks*

- When approaching the MhM, it can be difficult to properly identify its midline since the muscle fibers of both sides at this level are usually merged. An easy way for its correct identification is therefore to cut with electrocautery a line running from the genial tubercles to the midline of the HB identified by palpating the thyroid notch.
- Once the MhM is detached from the deeper GhM, particular care should be addressed to obtain accurate hemostasis due to the large number of small perforators between the two muscles. Keeping the surgical field as clean as possible in this phase may be of great help in correctly identifying the median raphe and contralateral muscular compartment.

### Step 6 - Deeper Midline Raphe Dissection

**Figure 7:**
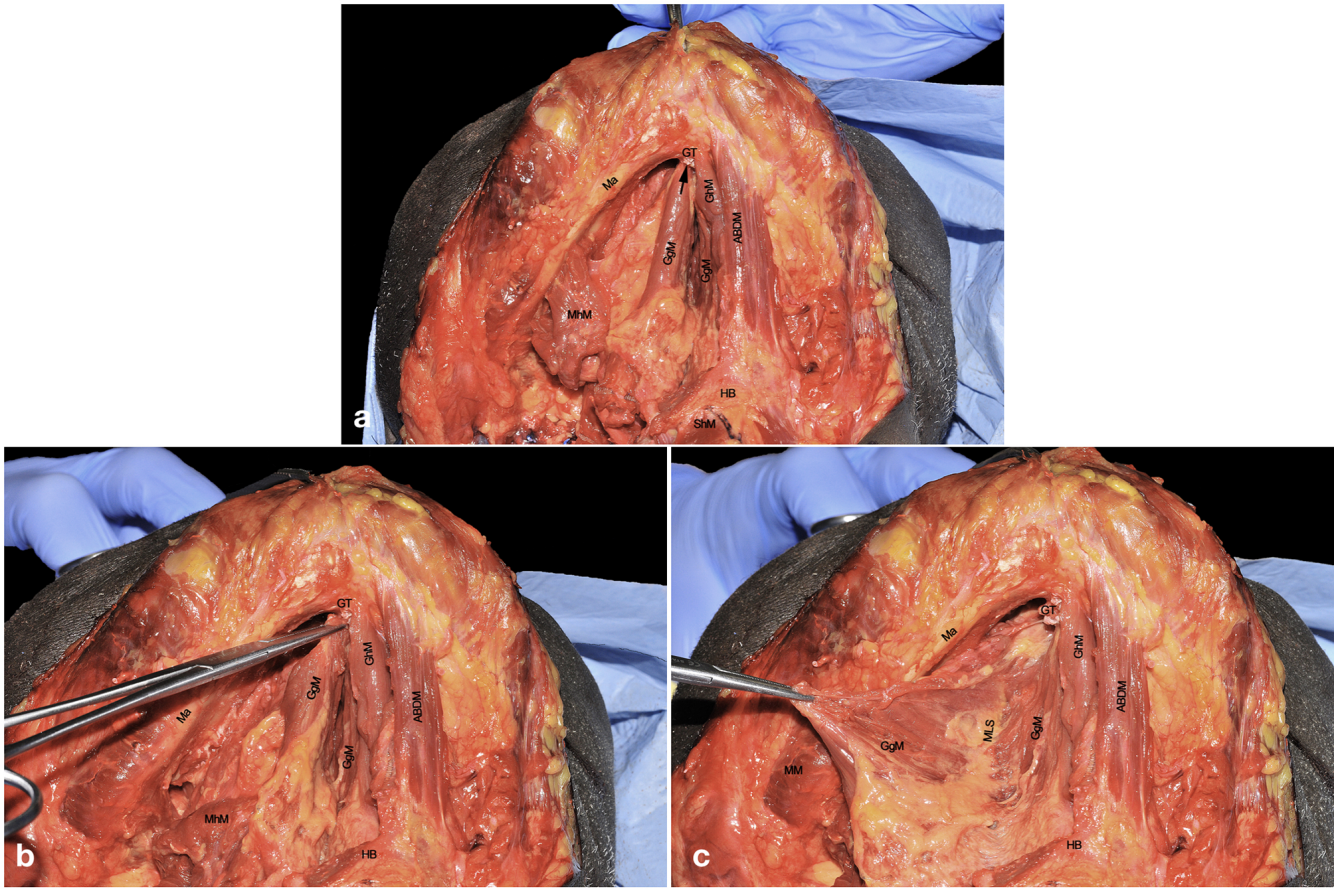
**(A–C)** ABDM, anterior belly of digastric muscle; GgM, genioglossus muscle; GhM, geniohyoid muscle; GT, genial tubercle; HB, hyoid bone; Ma, mandible; MhM, mylohyoid muscle; MM, masseter muscle; MLS, median lingual septum; ShM, sternohyoid muscle. Black arrow, right inferior genial tubercle.

Once the GhM has been resected, the midline fibrous raphe between the two hemilingual compartments is clearly recognizable. It is imperative to not resect muscular or neurovascular structures of the healthy side in order to minimize any undue functional impairment. Separation of the two compartments at this level is usually easily obtained by blunt dissection, using a cotton swab or dissector. This maneuver is also useful to palpate the lingual body from below in order to assess the tumor characteristics and possible critical issues such as endophytic extension, distance from the midline, or presence of satellitosis. The insertion of the removed right GhM is visible at the level of the inferior genial tubercle (arrow), while the deeper extrinsic muscular layer (represented by the GgM) is now clearly visible (**Figure 7A**). The latter is the most represented structure of the mobile tongue and is of pivotal importance for tumor spread due to its spatial fan-shaped fibers arrangement, disposed from the upper genial tubercle to the rest of the mobile tongue itself, and its large volume accounting for more than half of the lingual body. The GgM must be separated from the contralateral one using monopolar cautery, starting from its mandibular insertion, to the HB and glosso-epiglottic valleculae (**Figure 7B**).

**Figure 8:**
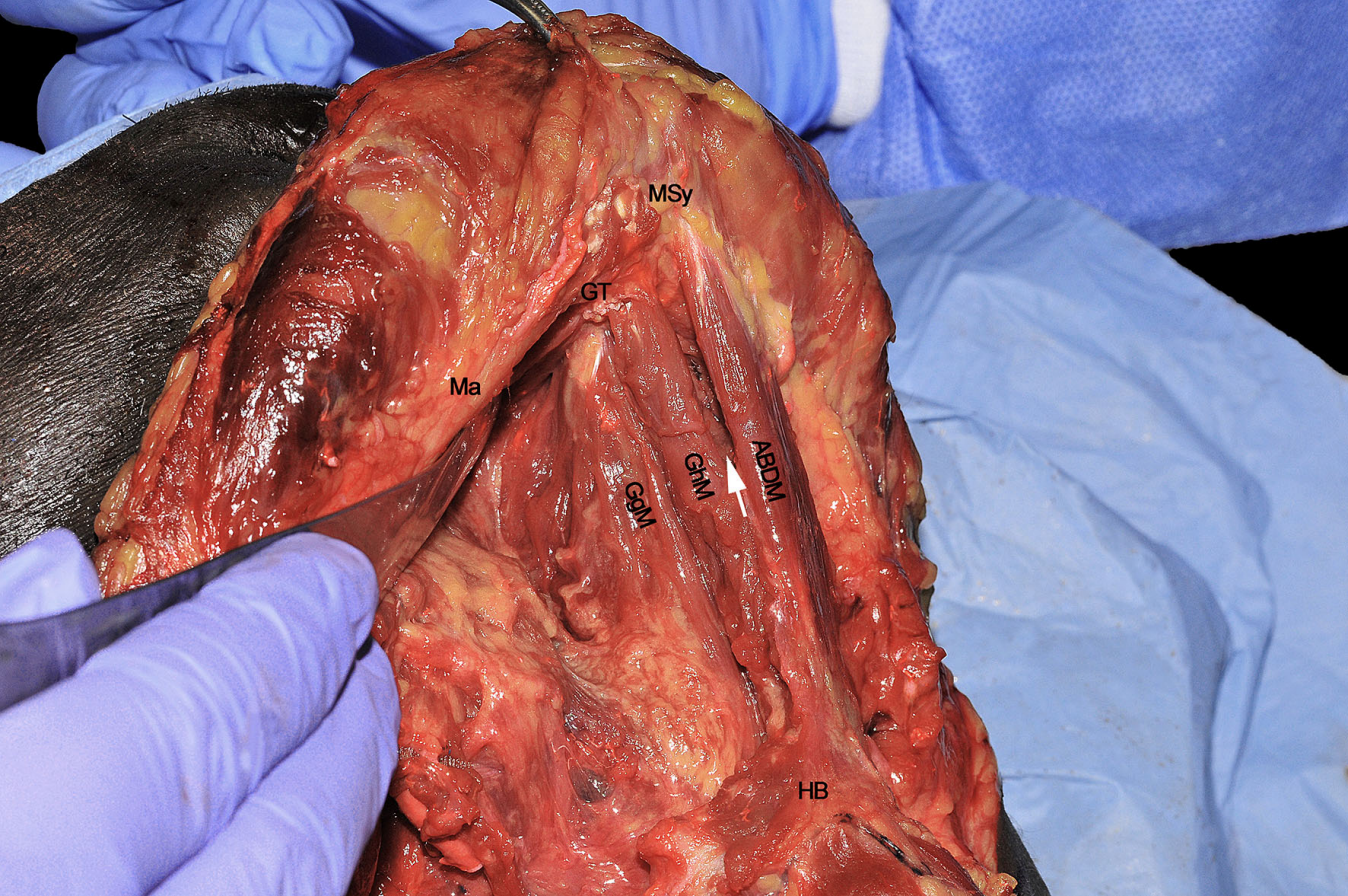
ABDM, anterior belly of digastric muscle; GgM, genioglossus muscle; GhM, geniohyoid muscle; GT, genial tubercle; HB, hyoid bone; Ma, mandible; MSy, mandibular symphysis. White arrow, thin left mylohyoid muscle between the ipsilateral ABDM and GhM.

The resected GgM is laterally and posteriorly displaced, pulling it from its mandibular tendon (**Figure 7C**). Both cranially and caudally, particular care should be paid to not damage the floor of mouth or glosso-epiglottic mucosa. In this way, the median raphe is completely transected up to the intrinsic tongue musculature and mucosa. When the midline dissection is complete, a clear vision of the opposite tongue compartment should be appreciated (**Figure 8**).

*Surgical tips and tricks*

- A useful trick to correctly identify the midline is to use a cotton swab to manipulate the muscular bundles and avoid annoying bleeding. The GgM and GhM are covered by a thin fascia that give these structures a round and well-defined contour, helping the surgeon to recognize the fascial planes between different muscular layers.
- When the midline is precisely located, the assistant surgeon should apply countertraction from the healthy side to facilitate dissection. Once the muscles are spread apart, the yellowish tissue of the median raphe is exposed. At this point, a gauze can be placed from below in order to keep one compartment separated from the contralateral one and to correctly identify the midline when subsequently performing the transoral resection by incising the mucosa between the two Wharton’s ducts.

### Step 7 – Assessment of Routes of Tumor Spread

**Figure 9:**
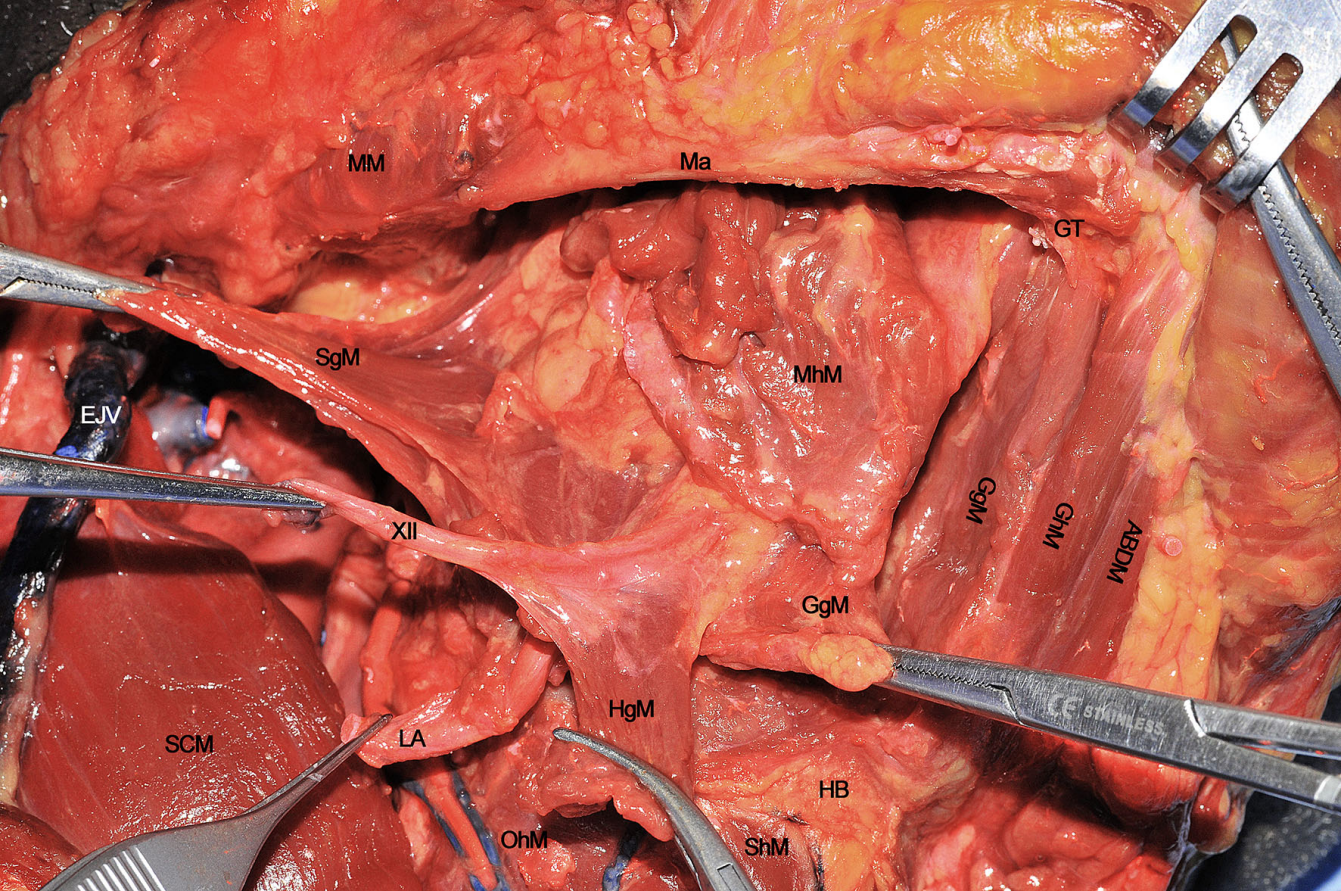
ABDM, anterior belly of digastric muscle; EJV, external jugular vein; GgM, genioglossus muscle; GhM, geniohyoid muscle; GT, genial tubercle; HB, hyoid bone; HgM, hyoglossus muscle; LA, lingual artery; Ma, mandible; MhM, mylohyoid muscle; MM, masseter muscle; OhM, omohyoid muscle; SCM, sternocleidomastoid muscle; SgM, styloglossus muscle; ShM, sternohyoid muscle; XII, hypoglossal nerve.

When every extrinsic muscular, neural and vascular structure have been dissected, the entire ipsilateral hemilingual compartment is detached from its anterior/cranial (mandible), caudal (HB), contralateral (opposite lingual compartment), and posterior/cranial insertions (mastoid and styloid processes). The most important advantage of CS is that it allows the most adequate control of all possible routes for centrifugal tumor spread that, starting from posterior to anterior, in an anti-clockwise direction, are shown herein: 1) SgM (towards the skull base), 2) XII cranial nerve (towards the vascular axis and skull base), 3) LA (towards the vascular axis), 4) HgM (towards the HB), and 5) GgM (towards the mandible) (**Figure 9**). In the middle of this complex surgical space, the MhM and GhM alongside the LN and all the lymphatic and glandular structures are located.

### Step 8 - Transoral Resection and Pull-Through Maneuver

**Figure 10:**
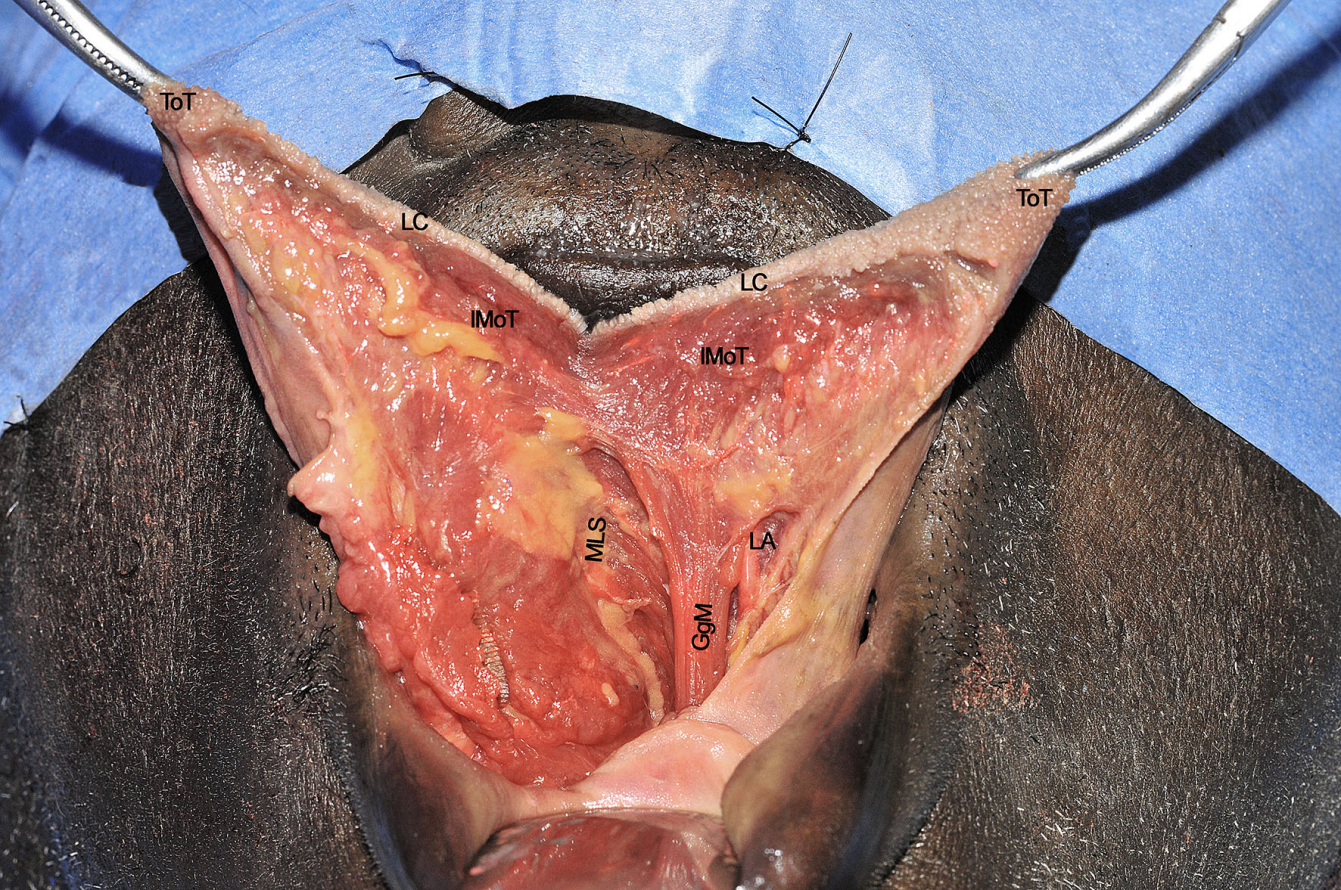
GgM, genioglossus muscle; IMoT, intrinsic musculature of the tongue; LA, lingual artery; LC, lingual cover; MLS, median lingual septum; ToT, tip of tongue. Please note that the nose is covered by the blue drape and the inferior lip pulled down by a retractor.

**Figure 11:**
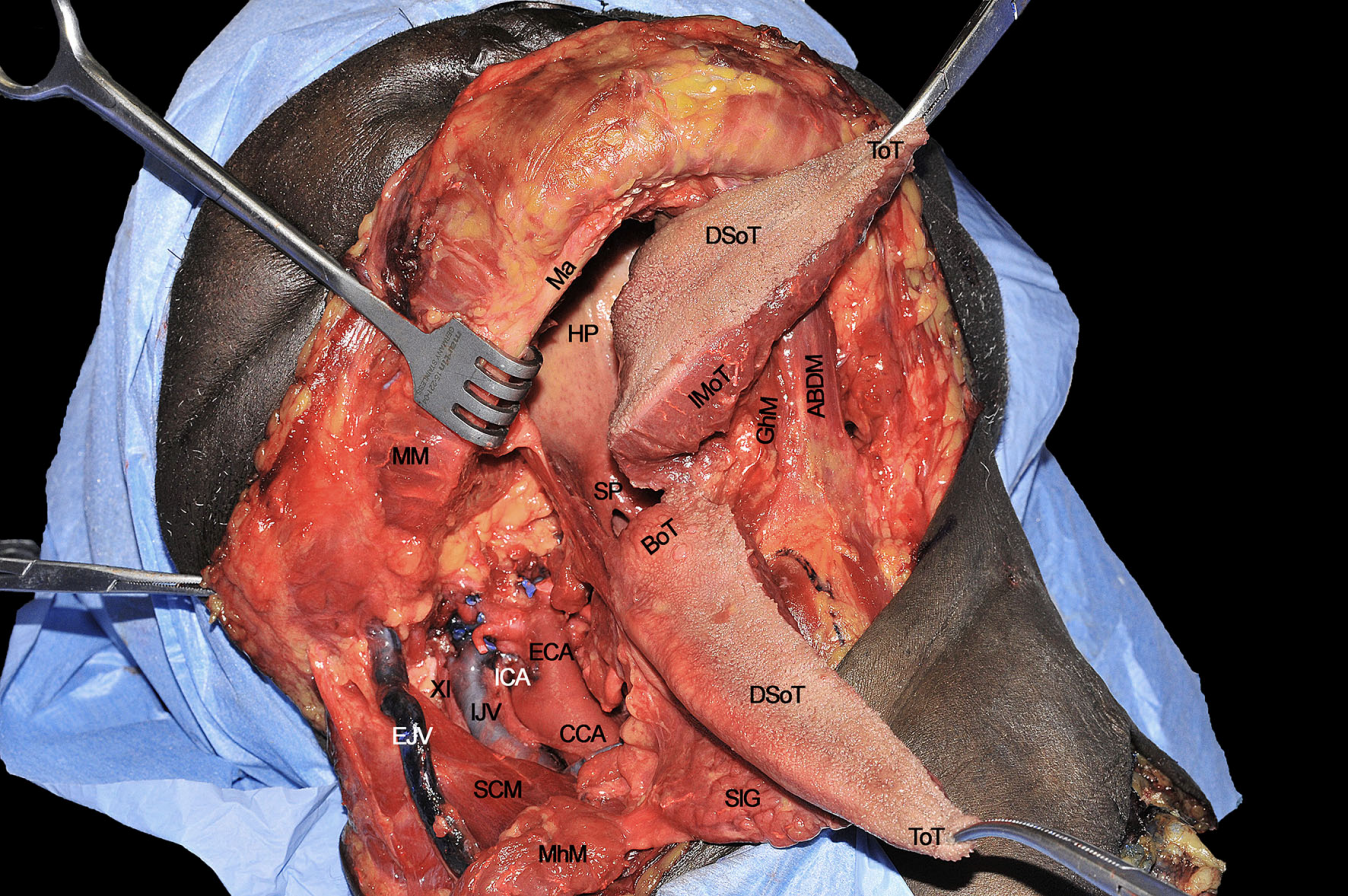
ABDM, anterior belly of digastric muscle; BoT, base of tongue; CCA, common carotid artery; DSoT, dorsal surface of tongue; ECA; external carotid artery; EJV, external jugular vein; IJV, internal jugular vein; GhM, geniohyoid muscle; HP, hard palate; ICA, internal carotid artery; IMoT, intrinsic muscle of tongue; Ma, mandible; MhM, mylohyoid muscle; MM, masseter muscle; SCM, sternocleidomastoid muscle; SlG, sublingual gland; SP, soft palate; ToT, tip of tongue; XI, spinal accessory nerve.

When the lingual compartment (right in the present cadaver dissection) has been prepared from the neck, it is possible to access the tumor through the oral cavity. The operating surgeon moves to the patient’s head and dissection starts from the anterior FoM, between the two openings of the Wharton’s ducts. A communication with the underlying neck space is created at this level and identification of the gauze previously positioned from the neck helps the surgeon to complete the dissection in the correct plane. Next, resection proceeds in a caudo-cranial direction, towards the ventral and dorsal surfaces of the tongue, maintaining the tumor under direct vision/palpation, and strictly following the midline lingual raphe. Dissection can be safely carried *via* the transoral route up to the circumvallate papillae in most patients without trismus (**Figure 10**). According to the posterior extension of the tumor towards the oropharynx, if the surgeon needs to perform a complete tongue base removal, the pull-through maneuver must be performed by cutting the mucosa of the lateral (paramandibular) FoM and pulling the involved hemilingual compartment into the neck. In this way, a complete compartmental resection addressing the posterior aspect of the base of tongue up to the glosso-epiglottic vallecula can be safely accomplished (**Figure 11**). Otherwise, if some mucosa and intrinsic muscles of the base of the tongue can be safely spared since a large (i.e. 2 cm) cuff of healthy tissues is already present at the posterior margin of the tumor, the dissection proceeds in an anterior to posterior direction reaching the hyo-glossal membrane, anterior and deep to the base of tongue, perpendicular to the midline raphe, where surgical resection may change its course from medial to lateral, up to the anterior tonsillar pillar. This surgical step is quite important since the entity of oropharyngeal resection can be modulated under direct view through the neck.

**Figure 12:**
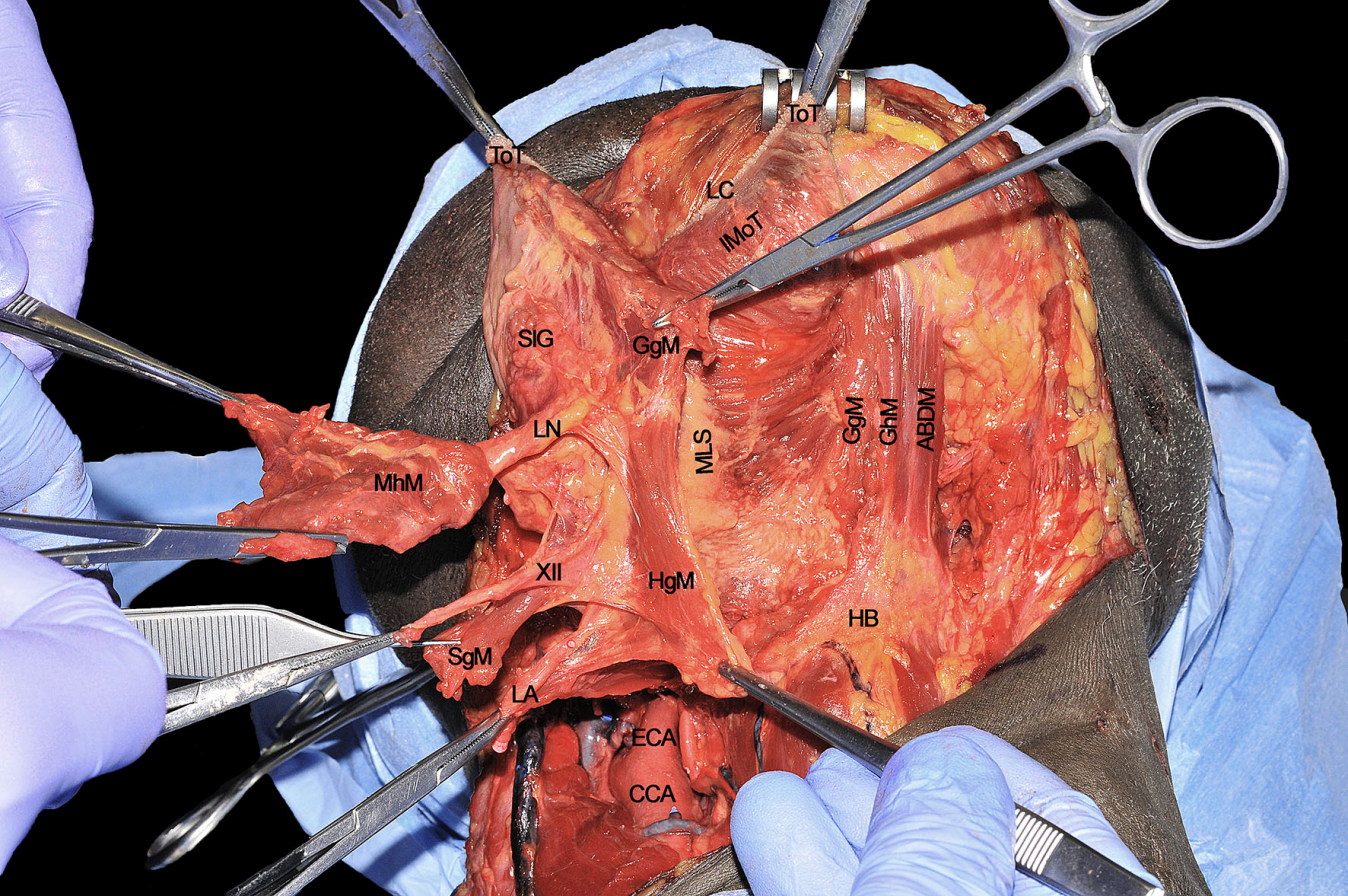
ABDM, anterior belly of digastric muscle; CCA, common carotid artery; ECA; external carotid artery; HB, hyoid bone; HgM, hyglossus muscle; GgM, genioglossus muscle; GhM, geniohyoid muscle; IMoT, intrinsic muscle of tongue; LA, lingual artery; LC, lingual cover; LN, lingual nerve; MhM, mylohyoid muscle; MLS, median lingual septum; SgM, styloglossus muscle; SlG, sublingual gland; ToT, tip of tongue; XII, hypoglossal nerve.

The T-N tract, completely released from all its muscular insertions and neural/vascular supplies, is visible as composed by the sublingual and submandibular glands (previously removed), extrinsic tongue muscles (GhM, GgM, HgM, and SgM) and MhM, LA and vein, XII cranial nerve, LN, and the lymphatic vessels and nodes embedded in the median and paramedian septa (**Figure 12**). The final step of the CS is represented by the detachment of the specimen from the glosso-epiglottic vallecula, keeping attention to spare healthy mucosa at this level which can be used for an effective flap suture during the reconstructive phase.

*Surgical tips and tricks*

- The tracheostomy (if not done previously) should be performed to remove the tube from the oral cavity and thus secure the postoperative airways. In some cases, this step can be postponed at the end of surgery if nasotracheal intubation has been performed. Others prefer to perform tracheostomy at the beginning of surgical procedure, to avoid any possible neoplastic seeding at the level of the tracheal wound by “contaminated” instrumentation. In any case, before starting the transoral step, the anesthesiologist should provide an optimal muscle relaxation in order to maximize oral opening.
- A self-retaining mouth and lips retractor and/or bite blocks can be used to optimize the view of the surgical field.
- Electrocautery or ultrasonic scalpels are usually applied to reduce bleeding during this phase, especially at the level of the contralateral (healthy) hemilingual compartment.
- The tip of tongue can be spared when not directly involved by the tumor since it is composed only of intrinsic muscles and not anatomically reached by the GgM fibers. This definitively improves postoperative speech without affecting oncologic outcomes.
- When sampling tissue for frozen sections, especially along the midline of the oral tongue, it is advisable to use a cool blade knife to reduce the shrinkage and cautery/crush artifacts of the specimen to be analyzed.
- When dissection between the two openings of the Wharton’s duct in the anterior aspect of the FoM is performed, particular attention should be paid to not accidentally injure the healthy side. The same holds true when suturing, at the end of surgery, the free flap at this level.
- Whenever the midline is reached by the tumor without invasion of the opposite side, a cuff of extrinsic/intrinsic muscles of the healthy hemilingual compartment should be taken as a safe extra-margin.
- When lateral FoM mucosa is incised medial to the mandibular body, at least 5 mm of healthy tissue should be spared at this level, if oncologically feasible, in order to guarantee enough recipient mucosa for the in-set of the free flap. If this is not possible, suturing the flap cutaneous edge to the mucosa of the gum passing the stitches in between teeth can be used as an effective trick to reduce the risk of salivary fistula.
- For tumors massively involving the BoT and/or associated with severe trismus, a median/paramedian mandibulotomy to widen the surgical field and guarantee an optimal visualization and dissection of the tumor may be of great help. All the steps herein described for CS can be equally performed *via* transoral or transmandibular routes.

### Step 9 - Surgical Defect and Specimen Evaluation

**Figure 13:**
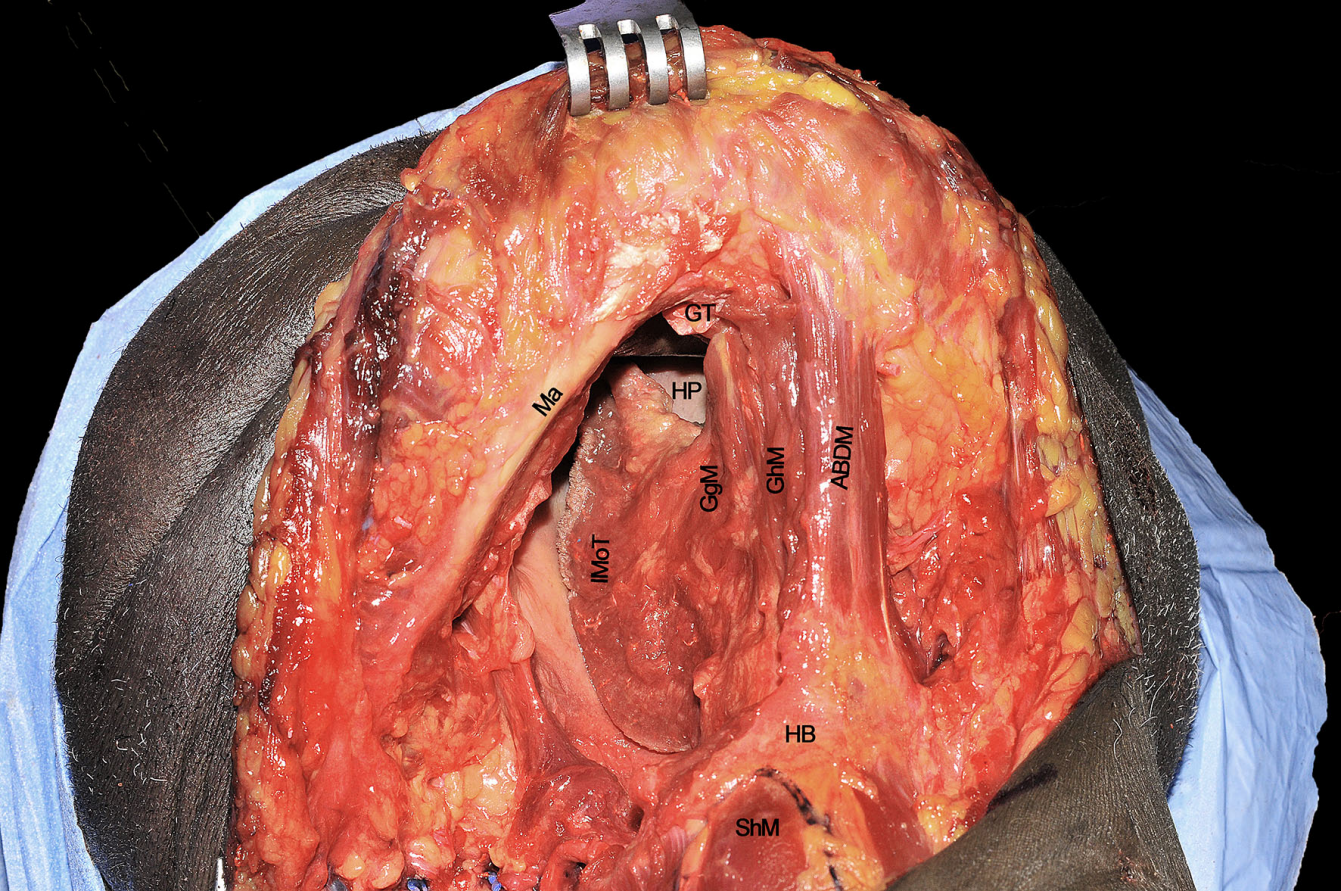
ABDM, anterior belly of digastric muscle; HB, hyoid bone; GgM, genioglossus muscle; GhM, geniohyoid muscle; GT, genial tubercle; HP, hard palate; IMoT, intrinsic muscle of tongue; Ma, mandible; ShM, sternohyoid muscle.

At the end of procedure, the surgical defect results in a wide communication between oral cavity/oropharynx and neck, delimited by the symphysis and body of the mandible anterolaterally, constrictor muscles and tonsillar region posteriorly, HB and glosso-epiglottic vallecula inferiorly, and healthy contralateral tongue compartment medially (**Figure 13**).

**Figure 14:**
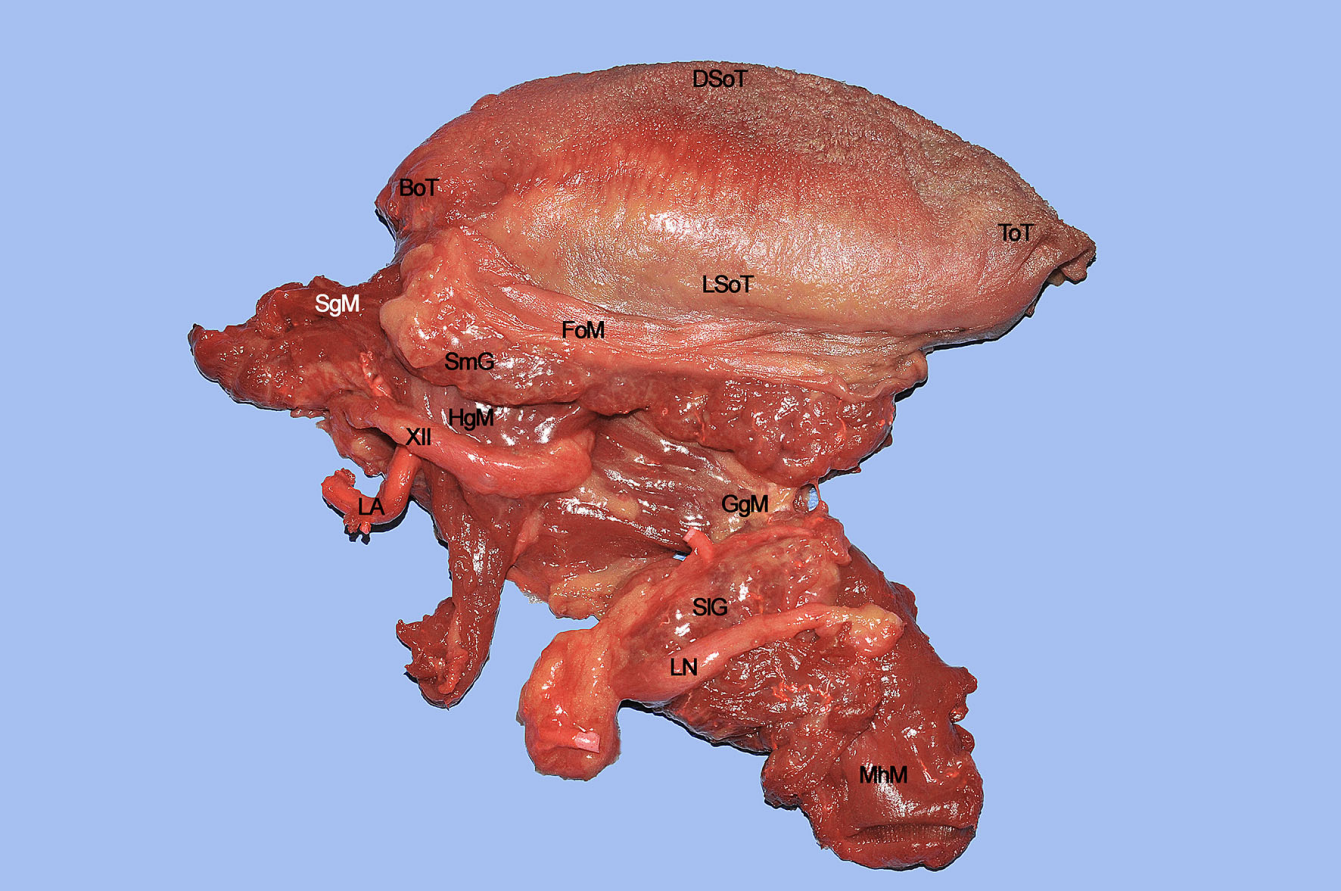
BoT, base of tongue; DSoT, dorsal surface of tongue; FoM, floor of mouth; GgM, genioglossus muscle; HgM, hyoglossus muscle; LA, lingual artery; LN, lingual nerve; LSoT, lateral surface of tongue; MhM, mylohyoid muscle; SgM, styloglossus muscle; SlG, sublingual gland; ToT, tip of tongue; XII, hypoglossal nerve.

The surgical specimen at the end of CS includes the right hemitongue (from the tip to the circumvallate papillae), ipsilateral FoM with related T-N tract, and the distal fringes of all the extrinsic muscles (**Figure 14**). The latter are herein clearly identifiable: the SgM reaches the tongue from posterior and its inferior muscular fibers intertwine in the posterior-lateral part of the tongue with those coming from the HgM, reaching the organ with an inferior-superior course. The LN runs in a lateral direction ventrally to the MhM adjacent to the SlG, while the XII cranial nerve is in strict relationships with the HgM, and the LA lies deeply to it. The fan-shaped fibers of the GgM are visible below the FoM and form large part of the volume of the tongue just cranially to the GhM.

## Discussion

CS of the tongue has been demonstrated to be a sound oncological technique, especially for tumors with a DOI ≥10 mm staged as cT3-T4 according to the 8th Edition of the AJCC-UICC TNM classification (13). The advantages are: 1) complete removal of the primary tumor along with the involved muscle compartment (which can potentially house in-transit perineural and/or lympho-vascular micrometastases and tumor satellitosis), thus improving local and loco-regional control; 2) surgical paradigm shift from a circumferential to an anatomical resection driven by the potential escape routes of the tumor itself; 3) standardization of the surgical ablation with consequent increase of both reproducibility and appropriate reconstructive planning.

This technique, first proposed by Calabrese and coworkers in 2009 (3), is based on the oncological principles of sarcoma surgery of the limbs (1, 2). In this view, the oral tongue is considered as a paired symmetric organ acting as the union of two compartments, identical in terms of spatial arrangement of extrinsic and intrinsic muscles and presence of intermuscular connective structures (median raphe, paramedian, and lateral septa) (12). The median raphe represents a natural barrier that separates each hemitongue from the opposite one, while laterally and anteriorly the mandibular periosteum represents its peripheral boundary. Inside the lingual body, all the extrinsic muscles, XII cranial nerve and LN, LA, veins and lymphatic vessels act as routes potentially causing persistent/recurrent loco-regional disease if not correctly addressed by surgery. This has been clearly demonstrated in seminal papers assessing the clinical relevance of the longitudinal path of tumor spread along tongue musculature (14, 15), quite similar to what has been described for sarcomas of the musculoskeletal system. Controlling such potential routes of tumor progression by a CS approach may significantly impact local and loco-regional tumor control as well as survival (4, 5, 16–19).

To support this thesis and anatomical considerations, Calabrese et al. in 2011 (4) presented the first case series of 143 patients affected by cT2-T4 cN0-N+ squamous cell carcinoma (SCC) of the oral tongue or floor of the mouth treated by CS compared to 50 patients treated by standard transoral surgery within clear margins (>1 cm). The oncological outcomes showed a 5-year local control (LC) of 88.4% (16.8% improvement compared to the control group), loco-regional control (LRC) of 83.5% (24.4% improvement), and overall survival (OS) of 70.7% (27.3% improvement).

Another study published in 2019 by Piazza et al. (5) focused on a retrospective analysis on 45 patients managed by CS for SCC of the oral tongue/floor of mouth (35 naïve patients and 10 in a salvage setting), showed that 2-year OS, disease free survival (DFS), LC, and LRC were 80%, 91%, 100%, and 94%, respectively, in previously untreated patients. On the other hand, prognosis was poor in those undergoing salvage surgery, showing a 2-year OS, DFS, LC, and LRC of 27%, 26%, 67%, and 36%, respectively. This confirmed that CS has a main role as primary surgical treatment in locally advanced oral tongue cancers, whereas survival remains extremely poor for recurrent disease.

The herein mentioned paradigm shift from a traditional circumferential to a CS approach can draw some criticisms because it can initially appear more aggressive, especially towards anatomical structures that are not macroscopically involved by the tumor. However, from a functional point of view, when a muscle is even partially resected as during standard surgery, its function is completely compromised and scar tissue substitutes it, potentially tethering the tongue residue, and impairing swallowing and speech functions. In fact, Ji et al. (20) demonstrated a significant difference between microsurgical reconstruction after partial *vs.* hemiglossectomies showing more functional impairment in the former. To further analyze functional outcomes in CS followed by microsurgical reconstructions in terms of swallowing and speech, a retrospective study on 48 patients was conducted by Grammatica and coworkers (7). The conclusion of this study was that CS does not significantly affect speech, while sub-clinical liquid food aspiration and vallecular pouch are present in a significant proportion of patients, especially after adjuvant non-surgical treatments. However, this issue is usually not subjectively perceived as a major problem, and no aspiration pneumonia occurred in our surgical series. Of note, when the residual tongue was tested using a device that objectively assesses muscular strength and endurance, it seemed that these were not macroscopically affected when proper reconstruction had been accomplished.

## Conclusions

The landmark concepts of CS herein depicted consist of: 1) anatomically-based approach to the lesion within the tongue and floor of mouth compartment, aiming to control all potential pathways of tumor progression; 2) clear identification of the anatomical space between primary and cervical nodes (the so called T-N tract), potentially acting as a high-risk metastatic basin; 3) good reproducibility and standardization of the surgical technique. A rational resectional approach associated with modern microsurgical reconstructive techniques maximizes oncological outcomes, while not affecting the mobility and function of the contralateral healthy hemitongue.

## Data Availability Statement 

The original contributions presented in the study are included in the article/supplementary material. Further inquiries can be directed to the corresponding author.

## Author Contributions

Study design: all authors. Anatomical dissections: AG, CP, MF, VV, LC. Drafting of the manuscript: all authors. Revision of the manuscript: AG, CP, AP, LC. All authors contributed to the article and approved the submitted version.

## Conflict of Interest

The authors declare that the research was conducted in the absence of any commercial or financial relationships that could be construed as a potential conflict of interest.
